# Low HDL cholesterol as a cardiovascular risk factor in rural, urban, and rural-urban migrants: PERU MIGRANT cohort study

**DOI:** 10.1016/j.atherosclerosis.2015.12.039

**Published:** 2016-03

**Authors:** María Lazo-Porras, Antonio Bernabe-Ortiz, Germán Málaga, Robert H. Gilman, Ana Acuña-Villaorduña, Deborah Cardenas-Montero, Liam Smeeth, J. Jaime Miranda

**Affiliations:** a CRONICAS Centre of Excellence in Chronic Diseases, Universidad Peruana Cayetano Heredia, Lima, Peru; b Unidad de Conocimiento y Evidencia, Universidad Peruana Cayetano Heredia, Lima, Peru; c Department of Medicine, School of Medicine, Universidad Peruana Cayetano Heredia, Lima, Peru; d Department of International Health, Bloomberg School of Public Health, Johns Hopkins University, Baltimore, USA; e Biomedical Research Unit, Asociación Benéfica PRISMA, Lima, Peru; f Faculty of Epidemiology and Population Health, London School of Hygiene and Tropical Medicine, London, United Kingdom

**Keywords:** HDL, Cholesterol, Human migration, Mortality, Myocardial infarction, Stroke

## Abstract

**Introduction:**

Whilst the relationship between lipids and cardiovascular mortality has been well studied and appears to be controversial, very little has been explored in the context of rural-to-urban migration in low-resource settings.

**Objective:**

Determine the profile and related factors for HDL-c patterns (isolated and non-isolated low HDL-c) in three population-based groups according to their migration status, and determine the effect of HDL-c patterns on the rates of cardiovascular outcomes (i.e. non-fatal stroke and non-fatal myocardial infarction) and mortality.

**Methods:**

Cross-sectional and 5-year longitudinal data from the PERU MIGRANT study, designed to assess the effect of migration on cardiovascular risk profiles and mortality in Peru. Two different analyses were performed: first, we estimated prevalence and associated factors with isolated and non-isolated low HDL-c at baseline. Second, using longitudinal information, relative risk ratios (RRR) of composite outcomes of mortality, non-fatal stroke and non-fatal myocardial infarction were calculated according to HDL-c levels at baseline.

**Results:**

Data from 988 participants, rural (n = 201), rural-to-urban migrants (n = 589), and urban (n = 199) groups, was analysed. Low HDL-c was present in 56.5% (95%CI: 53.4%–59.6%) without differences by study groups. Isolated low HDL-c was found in 36.5% (95%CI: 33.5–39.5%), with differences between study groups. In multivariable analysis, urban group (vs. rural), female gender, overweight and obesity were independently associated with isolated low HDL-c. Only female gender, overweight and obesity were associated with non-isolated low HDL-c. Longitudinal analyses showed that non-isolated low HDL-c increased the risk of negative cardiovascular outcomes (RRR = 3.46; 95%CI: 1.23–9.74).

**Conclusions:**

Isolated low HDL-c was the most common dyslipidaemia in the study population and was more frequent in rural subjects. Non-isolated low HDL-c increased three-to fourfold the 5-year risk of cardiovascular outcomes.

## Highlights

• Individuals from rural areas had greater prevalence of isolated low HDL-c compared to migrant and urban dwellers.
• Female gender, overweight and obese individuals had greater prevalence of isolated and non-isolated low HDL-c.
• Non-isolated low HDL-c was associated to greater mortality and cardiovascular events at follow-up.

## 1 Introduction

Cardiovascular disease-related deaths disproportionally affect low- and middle-income countries [1], and adverse lipid profiles have been linked to these events [2]. High-density lipoprotein cholesterol (HDL-c) is a significant predictor of major cardiovascular events, even after adjustment by other risk factors including baseline low-density lipoprotein cholesterol (LDL-c) level [3]. The role of HDL-c as a major contributor of myocardial infarction has been challenged [4] and current evidence supports a relationship between other biomarkers such as apolipoprotein B [5] and non-HDL-c cholesterol with coronary heart disease [6]. However, there is still data supporting an inverse relationship between HDL-c levels and risk of coronary heart disease [3], [7], and HDL-c remains a prevention cornerstone in major treatment guidelines [8].

It has been described that low HDL-c has two distinct patterns, 50% of cases have accompanying other dyslipidaemia and 50% have isolated low HDL-c, i.e. low HDL-c in the absence of other lipoprotein abnormalities [9]. Those with isolated low HDL-c have 20% additional risk of developing cardiovascular disease compared to people with normal levels of HDL-c [10]. On the other hand, other 21-year follow-up study did not find an association between isolated low HDL-c and coronary heart disease mortality when compared to people with high HDL-c [11]. These reports on the role of isolated low HDL-c as a major cardiovascular risk factor signal to an area of ongoing debate.

Several studies have reported prevalence of low HDL-c in different settings, yet very few have contrasted HDL-c profiles of rural versus urban populations [12], [13]. In the same vein, potential differences in HDL-c profiles based on migration status have not been thoroughly addressed, thus limiting opportunities to explore migration-related exposures resulting in lipid abnormalities. In an ever increasing urbanizing world [14], profiles of isolated/non-isolated low HDL-c and its relationship with migration, cardiovascular events and mortality remains to be characterised.

Migration studies intrinsically carry the potential to depict non-traditional risk profiles, and they are key positioned to inform avenues for further prevention and therapeutic targets. The aims of this study were twofold: first, to determine the profile of, as well as to characterize related factors for, HDL-c patterns with a focus on isolated and non-isolated low HDL-c in three population-based groups according to their migration status; and second, to determine the effect of HDL-c patterns on the rates of cardiovascular outcomes (i.e. non-fatal stroke and non-fatal myocardial infarction) and mortality.

## 2 Materials and methods

### 2.1 Study design, setting and participants

Data from the PERU MIGRANT study was analysed. Baseline assessment (conducted in 2007–2008) provided data for the cross-sectional analysis, and the follow-up assessment, after 5 years from baseline (2012–2013), was used for the longitudinal analysis.

The characteristics of the PERU MIGRANT study settings and enrolled participants have been described in detail elsewhere [15]. Briefly, a single-stage random sampling method was used in all study groups, and this procedure was stratified by age- and sex-groups. Rural participants were selected from the adult population permanently living in San Jose de Secker in Ayacucho (3239 m above sea level). Urban participants were born and currently live in Pampas de San Juan de Miraflores, a periurban shantytown in Lima, Peru, located at sea level. Rural-to-urban migrants were born in Ayacucho and lived in Pampas de San Juan de Miraflores.

### 2.2 Follow-up assessment

For re-evaluation, participants were re-contacted in the same setting where they were originally enrolled, thus re-confirming the rural, migrant and urban group status. Mortality and cardiovascular events, specifically non-fatal myocardial infarction and non-fatal stroke, were collected during the follow-up assessment. Death certificates were used to record date and cause of death. When death certificates were not available, especially in rural areas, the participant's relatives were interviewed to verify mortality and causes [16].

### 2.3 Variables definition

Low HDL-c was defined as HDL-c<40 mg/dL in men and <50 mg/dL in women [8]. For analyses purposes, three groups were created based on HDL-c values: i) normal HDL-c; ii) non-isolated low HDL-c, for instance low HDL-c accompanied by hypertriglyceridemia and/or high LDL-c (LDL-c ≥160 mg/dL) [8]; and iii) isolated low HDL, defined as low HDL-c without hypertriglyceridemia (triglycerides ≥200 mg/dL) and LDL-c<160 mg/dL. In addition, non-HDL-c was also calculated by subtracting HDL-c from total cholesterol, and then was categorised, defining ≥100 mg/dL as high non-HDL-c [8].

Other participant characteristics included in the analysis as related factors were age (in four categories, 30–39, 40–49, 50–59 and ≥ 60 years old) and gender. Socioeconomic status was analysed based on a deprivation index, a binary variable where “yes” refers to a participant in the bottom category of two or more of four deprivation variables: possessions weighted asset index (in tertiles), total family income per month (**<**US$150, US$150–250, **>**US$250 dollars), overcrowding (three or more people per room in house) and educational attainment (none/incomplete primary, complete primary, some secondary or higher). BMI categories (normal if BMI≥18.5 and < 25 kg/m^2^, overweight ≥25 and < 30 kg/m^2^, and obese ≥30 kg/m^2^) [17], current daily smoking (if a participant reported smoking at least one cigarette per day), heavy episodic alcohol intake (if participants reported drinking more than 6 servings of alcohol on one occasion), and physical activity levels. The later were categorised according to the International Physical Activity Questionnaire (IPAQ) as high/moderate if participant performed 5 or more days of any combination of walking, moderate-intensity, or vigorous–intensity activities achieving at least 600 MET (multiples of the resting metabolic rate) minutes/week or low if participant performed less than 600 MET minutes/week [18].

Blood pressure was measured three times and the average of the second and third measurements was used to define hypertension as systolic BP ≥ 140 mmHg or diastolic BP ≥ 90 mmHg, or self-report of physician diagnosis and currently receiving antihypertensive medication [19]. Fasting glucose was used to define diabetes if ≥ 126 mg/dL [≥7 mmol/L] or self-report of physician diagnosis and currently receiving anti-diabetic medication [20].

### 2.4 Laboratory procedures

Laboratory assessments were performed on venous samples taken in the morning after a minimum of 8 h fast. Serum glucose, HDL-c and triglyceride levels were measuring using a Cobas^®^ Modular Platform automated analyser and reagents supplied by Roche Diagnostics. The same procedure was used to measure LDL-c in people with triglycerides greater than 400 mg/dL (n = 29, 2.9% of all participants studied). In those with triglycerides below 400 mg/dL, LDL-c was calculated using the Friedewald equation in mg/dL [21].

### 2.5 Exposure and outcomes

For the cross-sectional analysis, the primary exposure of interest was study group, defined as rural, rural-to-urban migrant, and urban group.

For the longitudinal analysis the exposure of interest was HDL-c status at baseline (normal HDL-c, isolated low HDL-c, and non-isolated low HDL-c). Two set outcomes were pre-defined: first, mortality-only outcomes, i.e. all-cause mortality and cardiovascular mortality, and second, all-cause/cardiovascular mortality plus cardiovascular events, namely non-fatal myocardial infarction and non-fatal stroke.

### 2.6 Statistical analysis

All statistical analyses were performed using Stata 12.0 (StataCorp, College Station, TX, USA). Prevalence and 95% confidence intervals (95% CI) of different HDL-c categories were determined overall (frequency weighted) and by each study group. Chi-squared was used to address the relationship between sociodemographic and lifestyle factors with normal HDL, isolated low HDL-c and non-isolated low HDL.

Using baseline data, different models were generated using Poisson regression technique with robust variance as reported previously for cross-sectional data [22], [23] to find independently factors associated with our outcome of interest, and reporting prevalence ratios (PR) and 95% CI. Our first model allowed the comparison of isolated low HDL-c vs. normal HDL-c. A similar approach was used to compare non-isolated low HDL-c vs. normal HDL-c.

Using longitudinal data, crude and adjusted regression analysis was performed for modelling all composite outcomes, having HDL categories as exposure, and reporting relative risk ratios (RRR) and 95% CI. HDL-c classification as used in this manuscript allowed for the assessment of the independent risk of these profiles controlling for sex, age, deprivation index, and migration status. Adjustment for baseline LDL-c levels, as suggested in other longitudinal analysis of the effect of HDL-c on cardiovascular outcomes, was not pursued as normal (or abnormal) LDC-c level are part of the definition of isolated and non-isolated low HDL-c, already included in our definition of exposures.

### 2.7 Ethics

The Institutional Review Board of Universidad Peruana Cayetano Heredia in Peru approved the protocol for the baseline and follow up phase. All participants were informed about the objectives of the study and oral informed consent was obtained due to major illiteracy rates.

## 3 Results

Data from a total of 988 participants was included in the analyses. Of these, 201 (20.3%) were from the rural group, 588 (59.5%) from the rural-urban migrant group and 199 (20.1%) from the urban group. The mean age (SD) in rural, rural-to-urban migrant and urban areas was 48.3 (SD 13.1), 47.8 (SD 11.7) and 48.1 (SD 11.9) years, respectively. (See Supplementary Material 1).

### 3.1 Low HDL-c profiles in the study population

**Fig. 1:**
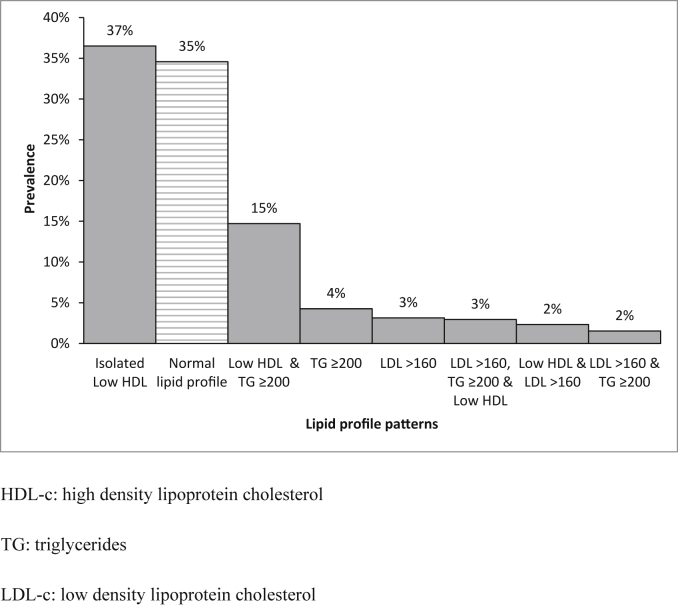
Lipid profiles in PERU MIGRANT Study at baseline.

Low HDL-c was present in 562/988 (56.5%; 95% CI 53.4%–59.6%), whereas isolated low HDL-c was present only in 387/988 (36.5%; 95% CI 33.5%–39.5%). In addition, high non-HDL-c was present in 843/988 (91.6%; 95% CI 89.8%–93.3%). Other combinations of lipid profile patterns are depicted in Fig. 1. Of note, none of the participants reported taking statins and only 3.7% reported to take antihypertensive drugs.

### 3.2 HDL-c patterns by sociodemographic and lifestyle factors

Isolated low HDL-c was present in 47.3% of rural, 38.1% of rural-urban migrants, and 34.2% of urban individuals (p = 0.02) with a pattern of gradients in prevalence between groups. The same pattern (rural > migrants > urban), but more marked, was present among females but not males. On the other hand, a different pattern, urban similar to migrant and both higher than rural, was observed in both high non-HDL-c and non-isolated low HDL-c (Table 1).

There was evidence of differences between normal HDL-c, isolated low HDL-c, and non-isolated low HDL-c profiles according to migration status, sex, age, deprivation index, BMI, and alcohol intake (Table 2).

### 3.3 Factors associated with low HDL-c profiles

In multivariable regression analyses, factors positively associated with higher prevalence of isolated low HDL-c were rural group compared to urban, female higher than males, and overweight and obese categories higher than normal BMI. On the other hand, factors positively associated with non-isolated low HDL-c included female gender, being 50–59 years old compared to those aged 30–39 years, as well as overweight and obese status relative to normal BMI. Although variables included in both models were similar (except migration status), the strength of association was higher when comparing non-isolated low HDL-c against normal HDL-c than those observed with isolated HDL-c (Table 3).

### 3.4 Mortality and cardiovascular events

Of the 988 participants enrolled at baseline in the PERU MIGRANT study, 895 were re-assessed, 60 (6.1%) were lost to follow-up, and 33 (3.3%) died. Of the total deaths with information on cause of death (n = 26), the number of deaths attributable to cardiovascular events was 9 (34.6%). In addition, a total of five (0.6%) individuals reported history of non-fatal myocardial infarction and 10 (1.2%) reported history of non-fatal stroke during the follow-up evaluation.

In multivariable models, subjects with non-isolated low HDL-c had three to four times more cardiovascular outcomes (RRR = 3.46; p = 0.02) than those in normal HDL-c group at baseline (Table 4). There was also a positive association between non-isolated low HDL-c and cardiovascular mortality (RRR = 3.66; p = 0.21), but its 95% CI span the value one.

## 4 Discussion

### 4.1 Main findings

In this study, we found that low HDL-c was the most frequent pattern of dyslipidaemia: it was present in nearly half of participants and, noticeably, isolated low HDL-c was present in almost 40% of the total study population. There were some notorious patterns of differences in prevalence between study groups depending of the HDL-c variable of interest.

In multivariable cross-sectional analyses, and relative to the urban subjects, rural individuals had more probability to have a higher prevalence of isolated low HDL-c but it was not the case for non-isolated low HDL-c. Finally, in longitudinal analyses, only non-isolated low HDL-c was associated with a three-to fourfold higher risk of cardiovascular endpoints using a composite outcome that included cardiovascular mortality, non-fatal myocardial infarction, and non-fatal stroke.

### 4.2 Prevalence of isolated and non-isolated low HDL-c

A systematic review assessing cardiovascular risk factors in urban, rural and migrant populations reported a gradient in which BMI, total cholesterol, LDL-c, and fasting blood glucose were higher in urban residents than migrants, and higher in migrants than rural dwellers, but evidence was weak to explore HDL-c in migrant versus non-migrant groups [24]. Our analysis showed a similar pattern of gradients among female subjects, but in the opposite direction, raising further questions about the role of low HDL-c in rural women, particularly in long-term outcomes.

Previous studies in Peruvian [25], [26], [27] and Latin American settings [28] have reported high overall rates of low HDL-c. Specifically for rural and high-altitude settings, other studies have shown low HDL-c frequencies varying from 45.3% to 77% [27], [29]. The high frequency of low HDL-c observed in Peru differs substantially when compared to other settings. For example, in the CARMELA study, a study of seven urban Latin American capitals and major cities, low HDL-c was reported as the most common dyslipidaemia of all lipid abnormalities [30]. In this study, Lima had the highest prevalence of low HDL-c (57%) much higher than the prevalence reported for Buenos Aires (16%), and Mexico City, Quito and Santiago de Chile (∼22%) [30]. Beyond the Latin American context, the prevalence of low HDL-c found in our study are higher than those reported in other studies [25], [26], [28], [29], [31], including Haiti (25%), France (30%), Portugal (36%), Italy (43%), and the Unites States (33%) [31], [32], [33].

### 4.3 Profile of isolated and non-isolated low HDL-c

Results from CARMELA's urban sites, using a cut-off point of 40 mg/dL to define low HDL in both sexes, showed that the frequency of low HDL-c was higher in males than females [30]. But, when rural sites are investigated, using the same definition for low HDL used in our analyses, this pattern is reversed, i.e. low HDL-c is more prevalent among females than males [26], [27], [34], in line with our findings. Our study expands in pointing out that much of this difference can be attributed to isolated low HDL-c.

Isolated low HDL-c was the more common dyslipidaemia in our study, with a prevalence varying from 34% in urban group to 47% in the rural group. In other settings, the prevalence of isolated low HDL-c appears to be much lower, e.g. 22% among Asians and 14% in New Zealand or Australia [13]. Nevertheless, rates showed in our study are similar to those found in India, where isolated low HDL-c was present in 45% of the overall population, with significant differences between urban (39%) and rural (47%) areas [12].

The high prevalence of low HDL-c observed merits some explanation, albeit difficult, as environmental or genetic factors could well play a role in the patterns observed. NHANES III data showed that the prevalence of low HDL-c in Mexican American women appears to be higher compared to US white or black females [35], but no differences were observed in males, pointing towards a genetic driver, particularly in women, as all groups share similar US-based environment. In another study, Mexican-Americans born in Mexico or in the US but currently residing in the US were found to have up to 80% lower chances of having low HDL-c relative to Mexicans in Mexico [36], introducing further challenges into the roles of genetics versus environment.

Overweight and obesity were consistently associated with isolated and non-isolated low HDL-c, but the magnitude of the association was stronger in the case of non-isolated HDL-c profile. Thus, whereas obesity increased almost 50% the probability of having isolated low HDL-c, this was 250% for non-isolated low HDL-c. It is well known that obesity causes hypertriglyceridemia, high LDL-c and low HDL-c, and of all of these adverse lipid profiles hypertriglyceridemia is the most frequent abnormality because of delayed clearance of the triglycerides-rich lipoproteins and formation of LDL-c [37]. For example, a study from China showed that participants with BMI ≥28 kg/m^2^ had 46% higher odds of having low HDL-c [38].

Diet is another important behaviour as HDL-c levels have been reported to be significantly lower in the highest tertile of carbohydrate intake than the lowest tertile, even after adjustment for obesity and other confounders [39]. Also, in societies in transition, there is some evidence that people from rural areas are consuming more carbohydrates than in urban settings [40], [41], [42], a plausible, albeit partial, explanation for our findings. Living at high altitude areas requires physiological adaptations, which in turn may provide additional avenues for explanations. Literature reports that higher HDL-c levels have been observed in high altitude compared to low-altitude groups, but this is contradictory to our findings [43].

### 4.4 Mortality and cardiovascular events

Low HDL-c has been extensively described as a risk factor for cardiovascular events and mortality. However, a recent study shows that an increase in 1 standard deviation due to genetic score −14 common single nucleotide polymorphisms associated to higher HDL-c levels— was not actually protective of myocardial infarction. Also, it has been reported that people with high HDL-c inevitably tend to have lower non-HDL-c [4]. As a result, current evidence support that high non-HDL-c is related to coronary heart disease and cardiovascular death [44], which in our setting raises further concern given the high prevalence of high non-HDL-c, up to 90%, reported in this study. We also found an increased risk of cardiovascular mortality, non-fatal stroke and non-fatal myocardial infarction among those with non-isolated low HDL-c, but not with isolated low HDL-c. Other study in Asians found isolated low HDL-c were as strongly associated with coronary heart disease risk (hazard ratio, 1.67 [95% CI, 1.27–2.19]) as non-isolated low HDL-c (1.63 [95% CI, 1.24–2.15]). However, this study did not find association with stroke and did not assess mortality as outcome [10].

The trajectories of individuals who shift locations and lifestyles across a diversity of scenarios connected to rural-urban migration processes, together with environmental complexities, e.g. low/high-altitude and/or rural/urban areas, will shape exposure patterns: who is exposed to what (combination of) factors, to what extent, and in which critical periods of life [45], [46]. Isolated and non-isolated HDL-c as a marker of long-term risk for hard outcomes requires a much finer understanding of these relationships, including the diversity of functionalities of the HDL-c [47]. Whilst physical activity is more common in rural people [48], urban residents tend to have greater economic advantage including higher education levels that could affect dietary quality or choices. Therefore, detailed attention to these patterns, both linked to HDL-c and lipid profiles in general, are required to pursue understanding of the relationship between HDL-c levels and cardiovascular risk.

### 4.5 Strengths and limitations

Some strengths and limitations deserve consideration. This study expands on previous knowledge about low HDL-c patterns in Latin American population taking advantage of a dataset that allowed comparisons according to migration status in rural/urban settings. However, other variables that may be related to HDL-c levels such as dietary patterns, contraceptive, thyroid hormone and hormone therapy use, or menopause were not addressed by the PERU MIGRANT study and could not be included in this analysis. Another limitation is the small number of deaths and cases with cardiovascular events (i.e. non-fatal myocardial infarction and non-fatal stroke), thus limiting the power for some analyses as evidenced by wide confidence intervals. Also, the cause of death comes from certificates and family report, and cardiovascular events were self-reported. As a result, some risk of misclassification might arise. Further studies are needed to corroborate our findings.

## 5 Conclusion

In summary, low HDL-c is very common and isolated low HDL-c was the predominant profile. Isolated HDL-c occurs more frequently in rural and female groups, and excess body weight is directly related to both isolated and non-isolated low HDL-c but with different magnitudes of association. Also, cardiovascular end points were only associated with non-isolated low-HDL-c. For these reasons to what extent low HDL-c contributes to the burden of cardiovascular disease in societies in transition merits attention, and migration studies can be well positioned to unveil distinct profiles through investigation of different human-gene-environment interactions, enabling newer avenues for further prevention and the identification of therapeutic targets.

## Grant support

The PERU MIGRANT Study baseline assessment work was funded through by a Wellcome Trust Master Research Training Fellowship and a Wellcome Trust PhD Studentship to JJM (GR074833MA), and its follow up by Universidad Peruana Cayetano Heredia (Fondo Concursable No. 20205071009). ML, AB-O, and JJM and the CRONICAS Centre of Excellence in Chronic Diseases were supported by Federal funds from the United States National Heart, Lung, and Blood Institute, National Institutes of Health, Department of Health and Human Services, under contract No. HHSN268200900033C. LS is a Wellcome Trust Senior Clinical Fellow (098504/Z/12/Z), and AB-O is a Wellcome Trust Research Training Fellow in Public Health and Tropical Medicine (103994/Z/14/Z).

## Figures and Tables

**Table 1:** Prevalence of low HDL-c by migration status and gender.

	Rural group (n=201)	Migrant group (n = 588)	Urban group (n = 199)	p-value
n, %, 95% CI	n, %, 95% CI	n, %, 95% CI	
**Low HDL-c**				
Total	113 (56.2%)	341 (57.9%)	108 (54.3%)	0.642
(49.1%–63.2%)	(53.9%–62.0%)	(47.1%–61.3%)
By sex				
Males (%)	33 (34.7%)	121 (43.4%)	44 (47.8%)	0.174
(25.3%–45.2%)	(37.5%–49.4%)	(37.3%–58.5%)
Females (%)	80 (75.5%)	220 (71.2%)	64 (59.8%)	0.031
(66.2%–83.3%)	(65.8%–76.2%)	(49.9%–69.2%)
**High non-HDL-c**				
Total	125 (62.2%)	533 (90.7%)	185 (92.9%)	<0.001
(55.1%–68.9%)	(87.9%–92.9%)	(88.5%–96.1%)
By sex				
Males (%)	56 (58.9%)	255 (91.4%)	89 (96.7%)	<0.001
(48.4%–68.9%)	(87.5%–94.4%)	(90.8%–99.3%)	
Female (%)	69 (65.1%)	278 (89.9%)	96 (89.7%)	<0.001
(55.2%–74.1%)	(86.1%–93.1%)	(82.3%–94.8%)	
**Isolated low HDL-c**				
Total	95 (47.3%)	224 (38.1%)	68 (34.2%)	0.019
(40.2%–54.4%)	(34.2%–42.2%)	(27.6%–41.2%)
By sex				
Males (%)	28 (29.5%)	72 (25.8%)	28 (30.4%)	0.61
(20.6%–39.7%)	(20.8%–31.4%)	(21.3%–40.9%)
Females (%)	67 (63.2%)	152 (49.2%)	40 (37.4%)	0.001
(53.3%–72.4%)	(43.4%–54.9%)	(28.2%–47.3%)
**Non-isolated low HDL-c**				
Total	18 (8.9%)	117 (19.9%)	40 (20.1%)	0.001
(5.4%–13.8%)	(16.7%–23.4%)	(14.8%–26.3%)
**By sex**				
Males (%)	5 (5.3%)	49 (17.6%)	16 (17.4%)	0.012
(1.7%–11.8%)	(13.3%–22.5%)	(10.3%–26.7%)
Females (%)	13 (12.3%)	68(22.0%)	24 (22.4%)	0.078
(6.7%–20.0%)	(17.5%–27.0%)	(14.9%–31.5%)

**Table 2:** Sociodemographic and lifestyle characteristics according to normal HDL, isolated low HDL-c and non-isolated low HDL-c.

	Normal HDL-c (n = 426)	Isolated low HDL-c (n = 387)	Non-isolated low HDL-c (n = 175)	p-value
n (%)	n (%)	n (%)
**Study group**				0.003
Urban	91 (45.7%)	68 (34.2%)	40 (20.1%)	
Migrant	247 (42.0%)	224 (38.1%)	117(19.9%)	
Rural	88 (43.8%)	95 (47.3%)	18 (8.9%%)	
**Sex**				<0.001
Female	158 (30.3%)	259 (49.6%)	105 (20.1%)	
Male	268 (57.5%)	128 (27.5%)	70 (15.0%)	
**Age**				<0.001
30–39 years	127 (45.0%)	126 (44.7%)	29 (10.3%)	
40–49 years	128 (45.4%)	98 (34.8%)	56 (19.8%)	
50–59 years	95 (34.9%)	106 (38.9%)	71 (26.1%)	
≥60 years	76 (50.0%)	57 (37.5%)	19 (12.5%)	
**Deprivation index**				0.028
No	307 (44.6%)	251 (36.5%)	130 (18.9%)	
Yes	119 (39.7%)	136 (45.3%)	45 (15.0%)	
**Daily smoking**				0.761
Yes	16 (48.5%)	11 (33.3%)	6 (18.2%)	
No	408 (42.8%)	376 (39.5%)	169 (17.7%)	
**Heavy Episodic Alcohol intake**				0.002
Low	373 (41.4%)	366 (40.6%)	162 (17.9%)	
High	53 (60.9%)	21 (24.1%)	13 (14.9%)	
**Physical activity**				0.341
Moderate/high	310 (42.7%)	293 (40.4%)	123 (16.9%)	
Low	110 (43.3%)	92 (36.2%)	52 (20.5%)	
**BMI**				<0.001
Normal	229 (55.6%)	149 (36.2%)	34 (8.3%)	
Overweight	142 (37.8%)	158 (42.0%)	76 (20.2%)	
Obese	54 (27.3%)	79 (39.9%)	65 (32.8%)	

**Table 3:** Factors associated with isolated low HDL-c and non-isolated low HDL-c.

	Isolated low HDL-c vs. normal HDL-c		Non-isolated low HDL-c vs. normal HDL-c	
Crude model	Multivariable model^a^	Crude model	Multivariable model^a^
PR (95% CI)	PR (95% CI)	PR (95% CI)	PR (95% CI)
**Study group**				
Urban	1 (Reference)	1 (Reference)	1 (Reference)	1 (Reference)
Migrant	1.11 (0.91–1.36)	1.11 (0.91–1.35)	1.05 (0.78–1.42)	1.09 (0.83–1.45)
Rural	1.21 (0.97–1.52)	**1.34 (1.01–1.77)**	**0.56 (0.34–0.91)**	0.86 (0.50–1.49)
**Sex**				
Male	1 (Reference)	1 (Reference)	1 (Reference)	1 (Reference)
Female	**1.92 (1.64–2.26)**	**1.75 (1.48–2.08)**	**1.93 (1.49–2.49)**	**1.49 (1.14**–**1.96)**
**Age**				
30 - 39 years	1 (Reference)	1 (Reference)	1 (Reference)	1 (Reference)
40–49 years	0.87 (0.71–1.06)	0.90 (0.75–1.09)	**1.64 (1.10–2.43)**	1.40 (0.95–2.06)
50–59 years	1.06 (0.88–1.27)	1.09 (0.92–1.29)	**2.30 (1.58–3.34)**	**1.90 (1.32–2.73)**
≥60 years	0.86 (0.68–1.09)	0.87 (0.69–1.09)	1.08 (0.64–1.81)	1.07 (0.64–1.78)
**Deprivation index**				
No	1 (Reference)	1 (Reference)	1 (Reference)	1 (Reference)
Yes	**1.19 (1.02–1.37)**	1.11 (0.91–1.35)	0.92 (0.69–1.23)	1.34 (1.02–1.75)
**BMI**				
Normal	1 (Reference)	1 (Reference)	1 (Reference)	1 (Reference)
Overweight	**1.34 (1.13**–**1.58)**	**1.45 (1.22**–**1.72)**	**2.70 (1.88**–**3.88)**	**2.54 (1.72**–**3.75)**
Obese	**1.51 (1.25–1.82)**	**1.45 (1.18**–**1.78)**	**4.23 (2.96**–**6.02)**	**3.32 (2.19**–**5.03)**
**Smoke daily**				
No	1 (Reference)	1 (Reference)	1 (Reference)	1 (Reference)
Yes	0.85 (0.54–1.35)	1.08 (0.69–1.68)	0.93 (0.46–1.87)	1.12 (0.56–2.22)
**Heavy Episodic Alcohol intake**				
Low	1 (Reference)	1 (Reference)	1 (Reference)	1 (Reference)
High	**0.57 (0.40–0.83)**	0.72 (0.51–1.03)	0.65 (0.39–1.08)	0.92 (0.53–1.61)
**Physical activity**				
Moderate/high	1 (Reference)	1 (Reference)	1 (Reference)	1 (Reference)
Low	0.94 (0.79–1.11)	0.95 (0.79–1.13)	1.12 (0.86–1.48)	0.94 (0.73–1.22)

Bold stands out some prevalences ratio or risk ratio with a significant confidence interval.

a Multivariable models were adjusted by all other variables listed in the table. Further adjustment for hypertension or diabetes status in each of these models did not alter the PR and 95% CIs (data not shown).

**Table 4:** Risk factors for mortality and composite outcome in the PERU MIGRANT study.

	All-cause mortality		Cardiovascular Mortality	
Bivariate model	Multivariable model^a^	Bivariate model	Multivariable model^a^
RRR (95% CI)	RRR (95% CI)	RRR (95% CI)	RRR (95% CI)
**HDL-c category**				
Normal HDL-c	1(Reference)	1(Reference)	1(Reference)	1(Reference)
Isolated low HDL-c	0.69 (0.33–1.49)	1.11 (0.49–2.51)	0.18 (0.02–1.49)	0.45 (0.05–4.24)
Non-isolated low HDL-c	0.44 (0.13–1.51)	0.82 (0.21–3.15)	0.83 (0.17–4.08)	3.67 (0.48–28.16)
	**All-cause mortality or non-fatal stroke or non-fatal myocardial infarction**		**Cardiovascular mortality or non-fatal stroke or non-fatal myocardial infarction**	
**HDL-c category**				
Normal HDL-c	1(Reference)	1(Reference)	1(Reference)	1(Reference)
Isolated low HDL-c	1.02 (0.54–1.95)	1.49 (0.77–2.89)	1.08 (0.41–2.87)	1.63 (0.68–3.92)
Non-isolated low HDL-c	1.07 (0.47–2.41)	1.69 (0.72–3.99)	2.19 (0.80–5.97)	**3.46 (1.23**–**9.74)**

Bold stands out some prevalences ratio or risk ratio with a significant confidence interval.

a Multivariable models were adjusted by sex, age, deprivation index, and migration status.
