# Supplementary material for: Low HDL cholesterol as a cardiovascular risk factor in rural, urban, and rural-urban migrants: PERU MIGRANT cohort study
Source: Atherosclerosis. 2016 Mar;246:36–43. doi: 10.1016/j.atherosclerosis.2015.12.039 (PMC4773291; doi:10.1016/j.atherosclerosis.2015.12.039)
Supplement: Supplementary file 1 [file mmc1.docx]

## Supplementary material 1. Sociodemographic and lifestyle variables by migration status

|  | **Rural group** | **Migrant group** | **Urban group** |
| --- | --- | --- | --- |
|  | **(n=201)** | **(n=588)** | **(n=199)** |
| **Sex** |  |  |  |
| Female | 106 (52.7%) | 309 (52.5%) | 107 (53.8%) |
| Male | 95 (47.3%) | 280 (47.5%) | 92 (46.2%) |
| **Age** |  |  |  |
| 30 - 39 years | 61 (30.4%) | 164 (27.8%) | 57 (28.6%) |
| 40 – 49 years | 56 (27.9%) | 173 (29.4%) | 53 (26.6%) |
| 50 – 59 years | 46 (22.8%) | 167 (28.4%) | 59 (29.7%) |
| ≥60 years | 38 (18.9%) | 85 (14.4%) | 30 (15.1%) |
| **Education** |  |  |  |
| None/some primary | 132 (65.7%) | 183 (31.1%) | 13 (6.6%) |
| Primary completed | 30 (14.9%) | 99 (16.8%) | 23 (11.6%) |
| Secondary or higher | 228 (19.4%) | 306 (52.1%) | 162 (81.8%) |
| **Deprivation index** |  |  |  |
| No | 21 (10.5%) | 482 (81.8%) | 186 (93.5%) |
| Yes | 180 (89.5%) | 107 (18.2%) | 13 (6.5%) |
| **Daily smoking** |  |  |  |
| Yes | 1 (0.5%) | 15 (2.6%) | 17 (8.5%) |
| No | 200 (99.5%) | 572 (97.4%) | 182 (91.5%) |
| **Alcohol intake** |  |  |  |
| Low | 178 (88.6%) | 542 (92.1%) | 182 (91.5%) |
| High | 53 (11.4%) | 47 (7.9%) | 17 (8.5%) |
| **Physical activity** |  |  |  |
| Moderate/high | 197 (98.1%) | 409 (70.3%) | 120 (60.6%) |
| Low | 4 (1.9%) | 173 (29.7%) | 78 (39.4%) |
| **BMI** |  |  |  |
| Normal | 195 (97.1%) | 465 (78.9%) | 131 (65.8%) |
| Overweight/Obese | 6 (2.9%) | 124 (21.1%) | 68 (34.2%) |
